# Slaughter Indicators, Carcass Measures, and Meat Quality of Lamb Fattened with Spelt (*Triticum aestivum* spp. *Spelta* L.)

**DOI:** 10.3390/foods10040726

**Published:** 2021-03-30

**Authors:** Josip Novoselec, Željka Klir Šalavardić, Danijela Samac, Mario Ronta, Zvonimir Steiner, Vinko Sičaja, Zvonko Antunović

**Affiliations:** Faculty of Agrobiotechnical Sciences Osijek, University of J.J. Strossamyer in Osijek, Trg Sv. Trojstva 3, 31000 Osijek, Croatia; zklir@fazos.hr (Ž.K.Š.); dsamac@fazos.hr (D.S.); mronta@fazos.hr (M.R.); zsteiner@fazos.hr (Z.S.); vinko-55@hotmail.com (V.S.); zantunovic@fazos.hr (Z.A.)

**Keywords:** spelt, lamb, carcass measures, meat quality, biochemical parameters, enzyme

## Abstract

The aim of this research was to investigate the slaughter indicators, carcass measures, and meat quality of lambs fattened with spelt. Lambs consumed various feed mixtures (1000 g day^−1^ lamb^−1^): I—control group; II—group with 10% dehulled spelt; III—group with 20% dehulled spelt. In the blood, the concentrations of minerals (calcium, phosphorus, magnesium, and iron), biochemical parameters (urea, glucose, total proteins, albumin, globulin, cholesterol, triglycerides, high-density lipoprotein, low-density lipoprotein, ß-hydroxybutyrate, non-esterified fatty acids, glutathione peroxidase, and superoxide dismutase), and hepatic enzymes (alanine aminotransferase, aspartate aminotransferase, alkaline phosphatase, gamma-glutamyltransferase, and creatine kinase) were determined. After slaughter, carcass development was measured. Samples of musculus semimembranosus were taken, of which the physical properties were analyzed. By analyzing the production properties of the lambs, we found that the slaughter characteristics of the lamb carcasses and the physical properties of the lamb meat as well as most biochemical indicators and enzymes did not significantly differ. The concentrations of albumin were increased in the groups with 10% and 20% spelt, as was the activity of alkaline phosphatase in the group with 20% spelt. The presented results indicate that spelt is an appropriate ingredient in the diet for weaned lambs.

## 1. Introduction

Among the various production systems, in recent years, the organic production system has gained considerable importance, with the best prospects for even greater development in the future. In the last decade, the percentage of organic agricultural land has doubled [1]. Of the organic products, the most widespread are fruits and vegetables, and the least so is meat of organic origin [2], but with significant growth and development characteristics. According to [3], the production of organic meat over the last six years increased by 42.96%, and that of organic lamb meat by 11.81%. In the Republic of Croatia, there was a significant increase in the number of organically reared sheep in the period 2013–2019, from 19,411 to 65,632 (number of heads) [4]. Many studies have found that consumers buy organic food because they see it as healthier, and its production takes care of animal welfare and the environment [2,5,6]. Although there is no scientifically based evidence that organic food products are more nutritious than conventional ones [7], the fact that various organic livestock feed exclusively on green mass (pastures) and organic pesticide-free feeds has reduced their exposure to residues and antibiotic-resistant bacteria [7,8]. A significant problem in organic husbandry is ensuring an optimal ration in the winter feeding season, when animals fed dry feed must have a certain amount of concentrate composed of cereals of organic origin at their disposal. Organic cereal cultivation is more expensive than conventional cultivation because a large share of human labor is required and yields are lower. Given the above, spelt (*Triticum aestivum* spp. *Spelta* L.), due to its resistance (each grain has its hull) and, at the same time, modest or almost no requirements for fertilization and care, is suitable for organic farming and is an adequate replacement for conventional cereals used in the feeding of sheep and lambs [9]. Spelt has limited use as fodder due to the hull on the grain, but after dehulling, it is widely used as an ingredient in feed mixtures and in the bakery industry [10]. Spelt does not tolerate mineral fertilizers because it settles down, and it is then impossible to harvest them with a combine harvester; it also has a reduced content of gluten and a high content of fiber, which contributes to its easier and better digestion. The nutritional value of spelt is similar to the nutritional value of wheat and contains most nutrients needed in human and animal nutrition such as proteins, unsaturated fatty acids, vitamins (A, C, and B groups), minerals (calcium, cobalt, iron, phosphorus, magnesium, manganese, potassium, copper, selenium, and sodium), and fiber [11,12,13]. Due to selenium (a part of the enzyme superoxide dismutase that catalyzes the dismutation of superoxide into oxygen and hydrogen peroxide), spelt can have a positive influence on antioxidant protection [14] in the defense of almost all cells exposed to aerobic metabolism. In the available literature, there is no research on fattening lambs with spelt. Therefore, the present research aimed to investigate blood indicators of nutritional and health status, slaughter indicators, carcass measures, and meat quality of lamb fattened with spelt (*Triticum aestivum* spp. *Spelta* L.) and to determine whether spelt is suitable as an ingredient in the feed mixture of lambs.

## 2. Materials and Methods

### 2.1. Design of Experiment and Treatments

The research was conducted on an organic family farm in the village of Gašinci, Osijek-Baranja County, Croatia (45°20′05′′ N, 18°18′59′′ E), which raises Merinolandschaf sheep exclusively for the production of lamb. The research was conducted with 21 weaned lambs, Merinolandschaf breed, at an average age of 95 days. The lambs were divided by gender (3 females; 4 males) in each group and had good health:

- Control group;
- Group with 10% dehulled spelt;
- Group with 20% dehulled spelt.

Lambs consumed hay and water ad libitum. The ingredients and chemical compositions of the feed mixtures are shown in Table 1. The lambs consumed a feed mixture (1000 g day^−1^) made of organic feedstuffs origin. In the production of the mentioned feed mixtures for lambs, a mineral premix was used (Panto Mineral) which is certified for use in organic sheep and lamb rearing. For determination of the crude protein content in feed samples, the Kjeldahl method was used [15]. The Weende method [16] was used for determination of the crude fiber content in feed samples. The lambs were reared and fed according to Council Regulation (EC) no. 834/2007 on organic production [17]. Animals used in this study were maintained in facilities approved by the Croatian Association for Accreditation of Laboratory Animal Care and in accordance with current regulations and standards issued by the Croatian Ministry of Agriculture. Each group of lambs was kept separately with no access to pasture. The experiment lasted for a total of 30 days; animals were weighed every 15 days—at the beginning of the experiment (1 day), in the middle (15 days), and at the end (30 days).

### 2.2. Collection and Analysis of Blood Samples

Lamb blood samples were collected at the same time when they were weighted (1, 15, and 30 days) from the jugular vein (10 mL) into sterile vacuum tubes (Venoject^®^, Sterile Terumo Europe, Leuven, Belgium). After taking the blood samples, serum was separated by centrifugation (10 min) at 1609.92× *g* and analyzed in an Olympus AU640 analyzer. In the blood serum, mineral concentrations (calcium—Ca; inorganic phosphorus—P; iron—Fe; magnesium—Mg), biochemical parameters (urea, glucose, total proteins, cholesterol, albumin, globulin, triglyceride, high-density lipoprotein (HDL), low-density lipoprotein (LDL), hydroxybutyrate (BHB), non-esterified fatty acids (NEFAs), glutathione peroxidase (GPX), and superoxide dismutase (SOD)), and hepatic enzyme activity (alanine aminotransferase—ALT; aspartate aminotransferase—AST; alkaline phosphatase—ALP; gamma-glutamyltransferase—GGT; creatine kinase—CK) were all determined using Olympus System Reagents (OSR), manufactured and distributed by Olympus Diagnostica GmbH (Irish branch), Lismeehan, Ireland. Content of globulin was determined as the difference between total protein and albumin.

### 2.3. Slaughter and Carcass Evaluation

Before slaughter, lambs were weighed on an automatic animal scale Kern EOS 150K50XL (Kern and Sohn, Balingen, Germany). After slaughter (classical method of bleeding by cutting large blood vessels in the neck—*vena jugularis externa* and *arteria carotis communis*) and exsanguination, the lambs’ skin was peeled off the carcasses, and abdominal (forestomach, stomach, spleen, intestine, and liver) and thoracic (trachea with the lungs and heart) cavity organs were then detached. All internal organs, skin, lower parts of the legs, and the carcasses themselves were weighed. Finally, standard development measurements (linear measure) of lamb carcasses were taken: the length of the carcass (carcass length 1—*os pubis to atlas*; carcass length 2—*os pubis* to first rib; carcass length 3—*os pubis* to last rib), the circumference of the carcass at chest and ham, the length of the hind legs (*tuber calcanei to tubercle ossis ischia*), and the hind leg circumference (the widest part).

### 2.4. Meat Quality Evaluation

Lamb meat samples (*musculus semimembranosus*) were taken from all the lambs immediately after carcass processing, while pH values and color were determined 45 min post-mortem. The pH was measured with a Mettler Toledo contact pH meter using the spear-tip penetrating probe electrode method, while the color of the meat was measured with a Minolta Chroma Meter CR-410 portable instrument (Minolta Camera Co. Ltd., Osaka, Japan) according to the standard CIE L*a*b* color system [18]. Dressing percentage was calculated as (weight before slaughter—carcass weight × 100). Water-holding capacity was measured by the method of [19]. Hue angle was calculated according to the formula H* = tan^−1^(b*/a*) × (180/π), and chroma using the formula C* = √ (a*^2^ + b*^2^).

### 2.5. Statistical Analysis

Mean values of the obtained research results of production and slaughter indicators as well as carcass development indicators and physical properties were calculated by the MEANS procedure in the computer program TIBCO Statistica^®^ 13.3.0. Data were analyzed by means of an ANOVA, using feeding treatment as a fixed effect. Mean values were compared using Tukey’s test and differences between the groups were declared significant at *p* < 0.05 or less. Effects of treatment (I—control group; II—group with 10% dehulled spelt; III—group with 20% dehulled spelt) times (age) as repetitions and their interaction in the experimental period on production results and blood biochemical indicators were analyzed using GLM repeated measures ANOVA. Where the analysis showed significant differences, the LSD post hoc test was performed.

## 3. Results and Discussion

From the analysis of the production traits and slaughter indicators of lambs (Table 2 and Table 3), it is visible that there were no significant differences (*p* > 0.05) under varying nutrition influences.

Similar results in organic breeding of lambs in Italy and Croatia, with no significant differences in production traits as well as slaughter indicators (Table 2 and Table 3), were determined in the feeding of lambs with feed mixture in which peas were used as a source of protein instead of soybean meal [20,21]. A slightly lower dressing percentage in Merinolandschaf lambs compared to the present research has determined [22]. The higher dressing percentage of lambs established in the present research may be due to the lower live body weight of the lambs compared to the abovementioned Merinolandschaf lambs, which is confirmed by the results of the research [23].

The study [24] which investigated the effect of the incorporation of spelt in the concentrate on calf performance reported no effect on daily weight gain (*p* = 0.970). Furthermore, the authors concluded that spelt can stimulate concentrate intake, but no major effect on animal performance during the entire rearing period was noted. The authors showed a higher spelt concentrate intake by Holstein-Friesian calves during several weeks after weaning, but not in Belgian Blue double-muscled calves because of their limited intake capacity. Spelt has a lower energy content then wheat and barley; it is assumed that spelt is less able to fulfill energy requirements because of the lower energy density of spelt concentrate. This result is in accordance with the previous findings of [25], indicating that lambs avoid diets with a deficit or an excess of energy compared to lamb feed with a more adequate ration. In a 90-day trial in which an equal weight of spelt replaced oats or corn and was fed as a growth supplement to diary heifers, those fed oats or spelt exhibited similar live weight increases, while those fed corn had greater (*p* < 0.05) gains [26].

Feeding treatment did not significantly influence (*p* > 0.05) the carcass development and physical properties of the lamb carcasses and meat (Table 4 and Table 5). The measures of lamb carcass development of the processed carcasses of the studied lambs are comparable with the results of the research in [22] on the same breed of lambs. A higher water-holding capacity (WHC) was established in the meat of lambs that had consumed feed mixture with spelt, but differences were not significant (*p* = 0.824). The content of water in meat products is one of the most important quality parameters for meat processors as it relates to the final yield [27] of the final product and is also important in terms of eating. Water-holding capacity has a great impact on quality attributes such as juiciness and tenderness [28,29,30], and, if extreme, weakens the sensory perception of the meat. A number of intrinsic and extrinsic factors affect the WHC of meat. The most important intrinsic factors are genotype and feeding of animals, which affect muscle characteristics directly [31]. According to [32], to control extreme water loss during processing, it is largely accepted that pH is a crucial factor for controlling the ability of meat to hold water. Similar WHC and pH values in lamb meat to those in the present research were established [21] in organic breeding where lambs consumed feed mixture with the addition of peas.

Meat from suckling lambs is paler than that from weaned lambs because of the low concentration of iron in ewe milk [33]. The color of lamb meat is crucial to ensuring customer appeal and strongly contributes to the value of the product. According to [34] and [35], for fresh lamb meat, when the redness (a*) and lightness (L*) values are equal to or exceed 9.5 and 34, respectively, on average, consumers will consider the meat color acceptable, which agrees with results of present study. The acceptability thresholds derived by [34] for lamb equated to a chromameter L* value of 34–35 and a redness (a*) value below 19. Similar findings of meat color indicators were reported, with no statistical difference among nutritional treatments determined [21,22]. Lamb carcass characteristics and meat quality parameters (such as pH, meat color, water-holding capacity, and meat toughness) are consequences and results of the feeding system [36,37]. Meat from grazing animals has often been associated with yellow fat [38,39] and dark, tough, and little-flavored meat [37].

The lambs’ blood biochemical and mineral (Table 6 and Table 7) indicators were in the reference range in all three groups [40,41,42], which indicates the quality of their nutrition, which is also shown in the determined production traits and slaughter indicators (Table 2 and Table 3). Results comparable with the present research have previously been obtained [21]. In the present research, with progress of the trial, the values of some indicators (Fe, urea, HDL, and LDL) approached the reference values. The increase in AST up to 120 days of age resulted from the combination of an increase in mass and muscle activity and an improvement in the endogenous production of this enzyme with the development of the animal [43,44,45]. Creatine kinase is a muscle-specific enzyme characterized as a very sensitive bioindicator of the degree of activity, damage, and/or muscular effort [46,47]. According to [48], CK has high intramuscular activity and sensitivity and might vary quickly after minimal damage. Instability of this enzyme even in the face of common activities of routine management, such as restraint and weighing of animals, or as a result of intramuscular injections, exercise, or physical effort has been reported [40]. Therefore, it is possible that the variations in the present study might have occurred due to subtle differences in movement and the blood collection. Increased activities of ALP in the blood plasma of all lambs, regardless of dietary treatment, was observed in [49]. The authors claimed that high values of this enzyme are considered to be physiologically normal in growing animals, which agrees with the present research. High values of ALP are probably due to a fast growth rate that results in leakage of the enzyme from the growing bones and intestines into the blood [50]. Glucose levels were slightly higher compared to the reference values. According to [51], glycemia in ruminants is not influenced much by feeding, since it is regulated by an efficient hormonal homeostatic mechanism that aims to keep its concentration constant. Despite the performance of this mechanism, in the neonatal period and during the growth phase, glycemia is greatly influenced by age [44,45] and is related to the intake of colostrum and milk, as well as to the maturation of the liver, pancreas, and enzymatic activities and to the adaptation of the organism to the extrauterine environment [52]. The cholesterol concentrations were somewhat lower compared to the reference values in all groups during the entire trial. Research [53,54] has shown that the type of dietary protein, especially lysine-to-arginine ratio and the sulfur-containing amino acids (methionine and cysteine), has been considered a factor influencing the cholesterol serum concentration in rabbits. As verified among the animals evaluated in the studies by [45,55], a decrease in total cholesterol concentrations with advancing age was shown as a consequence of the modifications to the diet during the first months of life. The concentrations of the total proteins and globulin (Table 7) in the blood of the lambs increased with advanced age. As the age increases, the concentration of total protein in the blood of sheep increases [47,56]. The high total protein content after birth can be a result of serum immunoglobulin content growth, and this demonstrates good alimentary canal absorption and has some effect on later clinical state [57]. Albumin concentrations also affect the total protein levels. Albumin is the main plasma protein synthesized by the liver and corresponds to approximately 35–50% of total serum proteins and is responsible for 80% of the colloid osmotic pressure [41]. Albumin concentration is influenced by dietary protein intake and is considered the most sensitive indicator for the determination of protein nutritional status in the long term, since changes in its concentrations are detected only after a minimum period of one month due to its low rate of synthesis and degradation [58]. The cholesterol concentration is influenced by the degree of stress [59]. Therefore, lower cholesterol with age might be expected from stress (particularly by weaning lambs).

The significant decrease in triglycerides with aging might have been caused by changes in feeding management, as well as by the improvement in hepatic maturation and the ability to metabolize lipids [60]. Spelt did not significantly (*p* > 0.05) affect the activity of the enzymes SOD and GPX, thus not affecting the antioxidant activity of lamb’s blood, but a trend of higher values is present in the lambs fed with spelt. NEFA reflects the magnitude of fat mobilization from fat stores in response to negative energy balance. In the present study, NEFA levels (ranged from 0.00 to 0.36 mmol L^−1^) during the whole trial were consistent with normal levels (NEFA, <0.45 mmol L^−1^) described by [41]. Lower NEFA values indicate that there is less fat mobilization in these animals. BHB concentration may be a useful indicator in monitoring the energy status of lambs. In the present study, the BHB concentrations ranged from 0.20 to 0.32 mmol L^−1^ (normal level of BHB: 0.2 to 0.7 mmol L^−1^ according to [41]). Values of BHB from 0.80 to 1.60 mmol L^−1^ indicate a negative energy balance. Based on values of BHB and NEFA, we can conclude that the lambs in this study have a satisfactory energy status.

## 4. Conclusions

Spelt (*Triticum aestivum* spp. *Spelta* L.) is an ancient wheat species with a higher resistance to harsh environmental influences than common wheat. Based on the results from the present research, it can be concluded that spelt is an appropriate ingredient in the diet of weaned lambs and can partially replace barley and oats with no negative effect on lambs’ growth and the quality of lamb meat. Furthermore, given the results obtained in the present research, spelt could be a potentially suitable feed in organic lamb rearing.

## Figures and Tables

**Table 1:** Ingredients and proximate analysis of feed mixtures and meadow hay.

Component (%)	Group			Hay
	I	II	III	
Corn	39	39	39	
Oats	10	5	0	
Barley	15	10	5	
Wheat flour	15	15	15	
Soybean meal girts	18	18	18	
Spelt	0	10	20	
Mineral premix *	3	3	3	
Chemical composition (g kg^−1^ DM)				
Dry matter	891.5	890.0	891.4	862.5
Crude protein	157.2	160.1	159.05	141.5
Crude fat	25.3	24.5	21.3	12
Crude fiber	45.6	45.6	36.1	255.2
Ash	58.35	57.05	59.5	83.0
NEM MJ kg^−1^	736.4	736.6	736.88	2.26

I—control group; II—10% dehulled spelt; III—20% dehulled spelt; * 1 kg of premix contains VIT. A = 1,000,000 IJ; VIT. D3 = 150,000 IJ; VIT. E = 1500 mg; VIT. K3 = 50 mg; VIT. B1 = 100 mg; VIT. B2 = 200 mg; VIT. B6 = 200 mg; VIT. B12 = 1 mg; Ca pantotenat = 500 mg; Niacin = 1000 mg; Choline chloride = 20,000 mg; FeSO_4_ = 4000 mg; CuSO_4_ = 800 mg; Mn-oxide = 3500 mg; Zn-sulphate = 5000 mg; Cobalt chloride = 20 mg; Mg-sulphate = 10,000 mg; Antiox. dibutylhydroxytoluene (BHT) = 10,000 mg; 10,000 mg; Potassium iodide = 80 mg.

**Table 2:** Production traits of lambs.

Indicator	Age, Days	Group			SEM	*p*-Value		
		I	II	III		Group	Age	A. × G.
		Mean	Mean	Mean				
Body weight [kg]	95	28.76	28.63	28.77	0.36	0.932	<0.01	0.912
	110	32.80	32.57	32.63	0.50			
	125	35.89	34.92	34.99	0.72			
	Daily weight gain, g							
Average (1st—15th day)		269.71	262.67	257.14	26.11	0.790	<0.01	0.891
Average (15th—30th day)		205.71	156.76	157.14	18.65			
Average (1st—30th day)		237.71	209.71	207.14	19.26			
Feed conversion, g DM/g gain, (1–30)		4.40	4.97	4.99	-	-	-	-

Mean—arithmetic mean; SEM—standard error of mean; A.—age; G.—group; I—control group; II—10% dehulled spelt; III—20% dehulled spelt.

**Table 3:** Slaughter indicators of lambs.

Indicators, kg	Group			SEM	*p*-Value
	I	II	III		
	Mean ± sd	Mean ± sd	Mean ± sd		
Live weight at slaughter	35.89 ± 3.18	34.81 ± 4.42	34.67 ± 2.58	0.731	0776
Hot carcass weight	19.27 ± 1.75	19.53 ± 2.10	19.05 ± 0.97	0.349	0.864
Dressing percentage, %	53.52 ± 1.39	56.25 ± 2.14	55.05 ± 2.48	0.484	0.096
Organs *	1.79 ± 0.48	1.54 ± 0.28	1.63 ± 0.26	0.077	0.417
Forestomach and intestines	7.92 ± 0.80	6.74 ± 1.11	6.97 ± 1.07	0.237	0.092
Skin and lower legs	4.99 ± 0.38	5.04 ± 0.86	4.94 ± 0.47	0.126	0.952

Mean—arithmetic mean; sd—standard deviation; SEM—standard error of the mean; * lungs, trachea, heart, and liver; I—control group; II—10% dehulled spelt; III—20% dehulled spelt.

**Table 4:** Measures of lamb carcass development.

Indicators, cm	Group			SEM	*p*-Value
	I	II	III		
	Mean ± sd	Mean ± sd	Mean ± sd		
Carcass length ^1^	76.00 ± 2.58	75.14 ± 2.73	73.79 ± 3.51	0.647	0.391
Carcass length ^2^	55.71 ± 1.91	52.36 ± 4.31	52.79 ± 4.57	0.852	0.224
Carcass length ^3^	28.29 ± 2.12	29.35 ± 1.95	29.93 ± 2.82	0.505	0.424
Carcass circumference at chest	68.21 ± 2.41	68.21 ± 1.97	69.07 ± 1.33	0.406	0.634
Carcass circumference at ham	54.14 ± 2.98	54.79 ± 2.98	55.64 ± 2.43	0.597	0.613
Hind leg circumference	34.64 ± 3.70	33.21 ± 2.12	34.29 ± 4.81	0.726	0.726
Hind leg length	31.14 ± 1.38	32.00 ± 1.50	31.21 ± 1.58	0.319	0.501

Mean—arithmetic mean; sd—standard deviation; SEM—standard error of the mean; ^1^ carcass length (*pubis-atlas axis)*; ^2^ carcass length (*pubis axis*-first rib); ^3^ carcass length (*pubis axis*-posterior rib); I—control group; II—10% dehulled spelt; III—20% dehulled spelt.

**Table 5:** Physical properties of lamb meat.

Indicators	Group			SEM	*p*-Value
	I	II	III		
	Mean ± sd	Mean ± sd	Mean ± sd		
pH_1_	6.59 ± 0.31	6.58 ± 0.17	6.59 ± 0.18	0.047	0.992
WHC (%)	18.38 ± 5.39	20.05 ± 4.67	20.05 ± 6.97	1.205	0.824
Meat Color					
Lightness	35.58 ± 1.35	35.33 ± 1.74	34.92 ± 2.23	0.379	0.787
Redness	15.15 ± 0.92	14.63 ± 1.05	14.22 ± 0.81	0.211	0.198
Yellowness	0.55 ± 0.63	0.85 ± 0.59	0.61 ± 0.42	0.118	0.568
Hue angle	2.05 ± 2.29	3.22 ± 2.09	2.42 ± 1.59	0.432	0.551
Chroma	15.17 ± 0.92	14.66 ± 1.08	14.24 ± 0.82	0.214	0.208

Mean—arithmetic mean; sd-standard deviation; SEM—standard error of the mean; WHC—Water-holding capacity; I—control group; II—10% dehulled spelt; III—20% dehulled spelt.

**Table 6:** Blood enzyme indicators in lambs.

Indicators, UL^−1^	Age Days	Group			SEM	*p*-Value		
		I	II	III				
		Mean	Mean	Mean		Group	Age	G. × A.
AST	90	107.13	112.99	113.29	5.55	0.834	0.374	0.993
	105	101.80	102.99	100.59	3.64			
	120	110.11	110.90	115.03	5.35			
ALT	90	12.60	12.72	13.90	0.82	0.793	0.897	0.898
	105	13.41	14.10	12.96	0.82			
	120	12.57	14.59	13.89	0.73			
ALP	90	187.33	168.41	244.87	14.37	<0.001	<0.001	0.816
	105	333.07 ^a^	331.64 ^a^	482.40 ^b^	26.22			
	120	315.36	315.34	429.61	27.36			
GGT	90	59.27	73.00	64.11	4.15	0.553	0.476	0.538
	105	64.54	67.64	70.19	3.25			
	120	59.09	61.70	67.47	2.76			
CK	90	213.14	187.14	244.00	14.28	0.308	<0.001	0.490
	105	137.14	167.14	176.57	13.49			
	120	121.71	141.57	134.14	7.35			

Mean—arithmetic mean; SEM—standard error of the mean; G.—group; A.—age; I—control group; II—10% dehulled spelt; III—20% dehulled spelt; ^a,b^ (*p* < 0.05); AST—aspartate aminotransferase; ALT—alanine aminotransferase; ALP—alkaline phosphatase; GGT—gamma-glutamyltransferase; CK—creatine kinase.

**Table 7:** Blood biochemical indicators in lambs.

Indicators, Mmol L^−1^	Age Days	Group			SEM	*p*-Value		
		I	II	III				
		Mean	Mean	Mean		Group	Age	Gr. × A.
Mg	90	0.91	0.88	1.01	0.04	0.234	<0.001	0.947
	105	1.15	1.17	1.29	0.05			
	120	1.13	1.22	1.27	0.04			
Fe, µmol/L	90	19.60	25.70	25.39	2.06	0.133	0.011	0.902
	105	25.11	33.06	30.91	1.99			
	120	30.99	32.93	32.17	1.63			
P	90	2.61	2.37	2.59	0.10	0.806	0.001	0.421
	105	3.19	3.38	3.23	0.08			
	120	2.99	2.78	2.72	0.07			
Ca	90	2.42	2.41	2.36	0.03	0.621	<0.001	0.542
	105	2.57	2.59	2.64	0.03			
	120	2.61	2.53	2.63	0.02			
GUK	90	5.21	5.39	5.53	0.07	0.099	0.047	0.668
	105	5.50	5.75	5.84	0.11			
	120	5.42	5.18	5.54	0.09			
Urea	90	2.12	1.83	1.71	0.24	0.699	<0.001	0.016
	105	6.79	7.98	6.98	0.22			
	120	6.74	6.19	7.85	0.28			
PROT, gL^−1^	90	64.94	65.19	62.53	1.07	0.581	<0.001	0.869
	105	70.86	69.11	68.21	1.18			
	120	69.91	70.37	70.47	0.86			
ALB, gL^−1^	90	27.10	29.11	29.25	0.52	0.048	< 0.001	0.925
	105	29.60	30.86	30.72	0.39			
	120	30.11 ^a^	31.64 ^b^	31.90 ^b^	0.32			
GLOB, gL^−1^	90	37.84	36.07	33.26	1.09	0.166	0.036	0.855
	105	41.25	38.26	37.45	1.12			
	120	39.80	38.73	38.57	0.92			
CHOL	90	1.79	1.72	1.71	0.09	0.390	<0.001	0.976
	105	1.28	1.29	1.14	0.04			
	120	1.34	1.28	1.23	0.04			
TGC	90	0.42	0.29	0.34	0.02	0.094	0.001	0.575
	105	0.30	0.26	0.27	0.02			
	120	0.29	0.25	0.26	0.02			
HDL	90	1.16	1.07	1.12	0.05	0.337	<0.001	0.523
	105	0.82	0.96	0.75	0.05			
	120	0.86	0.80	0.82	0.02			
LDL	90	0.44	0.52	0.44	0.04	0.303	0.004	0.985
	105	0.32	0.35	0.27	0.02			
	120	0.35	0.37	0.30	0.03			
NEFA	90	0.36	0.22	0.13	0.08	0.538	0.005	0.691
	105	0.11	0.03	0.03	0.02			
	120	0.00	0.03	0.01	0.003			
BHB	90	0.32	0.23	0.25	0.025	0.125	0.612	0.399
	105	0.25	0.30	0.26	0.017			
	120	0.28	0.20	0.24	0.019			
GPX	90	336.09	298.07	332.74	26.19	0.979	0.260	0.886
	105	250.03	284.89	266.86	19.19			
	120	298.32	320.22	290.70	20.53			
SOD	90	0.33	0.23	0.32	0.03	0.365	0.046	0.609
	105	0.34	0.31	0.41	0.02			
	120	0.36	0.42	0.43	0.04			

Mean—arithmetic mean; sd—standard deviation; SEM—standard error of the mean; I—control group; II—10% dehulled spelt; III—20% dehulled spelt; ^a,b^ (*p* < 0.05); Mg—magnesium; Fe—iron; P—inorganic, phosphorus; Ca—calcium; GUK—glucose; PROT—total proteins; ALB—albumin; GLOB—globulin; CHOL—cholesterol; TGC—triglyceride; HDL—high-density lipoprotein; LDL—low-density lipoprotein; BHB—hydroxybutyrate; NEFA—non-esterified fatty acid; GPX—glutathione peroxidase; SOD—superoxide dismutase.

## Data Availability

The data presented in this study are available on request from the corresponding author.
